# Bioinspired Extracellular Vesicles: Lessons Learned From Nature for Biomedicine and Bioengineering

**DOI:** 10.3390/nano10112172

**Published:** 2020-10-30

**Authors:** Assaf Zinger, Ava Brozovich, Anna Pasto, Manuela Sushnitha, Jonathan O. Martinez, Michael Evangelopoulos, Christian Boada, Ennio Tasciotti, Francesca Taraballi

**Affiliations:** 1 Center for Musculoskeletal Regeneration, Houston Methodist Research Institute, Houston, TX 77030, USA; ABrozovich@houstonmethodist.org (A.B.); annapasto.phd@gmail.com (A.P.); msushnitha@houstonmethodist.org (M.S.); jmart1184@gmail.com (J.O.M.); michael.evangelopoulos@gmail.com (M.E.); christianboada@tamu.edu (C.B.); tasciottiennio@gmail.com (E.T.); 2 Department of Orthopedics and Sports Medicine, Houston Methodist Hospital, Houston, TX 77030, USA; 3 Texas A&M College of Medicine, Bryan, TX 77807, USA; 4 Department of Inflammation and Immunology, Humanitas Clinical and Research Center, 20089 Rozzano, Italy; 5 Department of Bioengineering, Rice University, Houston, TX 77030, USA; 6 Biotechnology Program, San Raffaele University, Via di Val Cannuta, 247, 00166 Roma RM, Italy

**Keywords:** cell communication, extracellular vesicles, biomimicry, drug delivery, biomedicine, bioengineering

## Abstract

Efficient communication is essential in all layers of the biological chain. Cells exchange information using a variety of signaling moieties, such as small molecules, proteins, and nucleic acids. Cells carefully package these messages into lipid complexes, collectively named extracellular vesicles (EVs). In this work, we discuss the nature of these cell carriers, categorize them by their origin, explore their role in the homeostasis of healthy tissues, and examine how they regulate the pathophysiology of several diseases. This review will also address the limitations of using EVs for clinical applications and discuss novel methods to engineer nanoparticles to mimic the structure, function, and features of EVs. Using lessons learned from nature and understanding how cells use EVs to communicate across distant sites, we can develop a better understanding of how to tailor the fundamental features of drug delivery carriers to encapsulate various cargos and target specific sites for biomedicine and bioengineering.

## 1. Introduction

**Figure 1:**
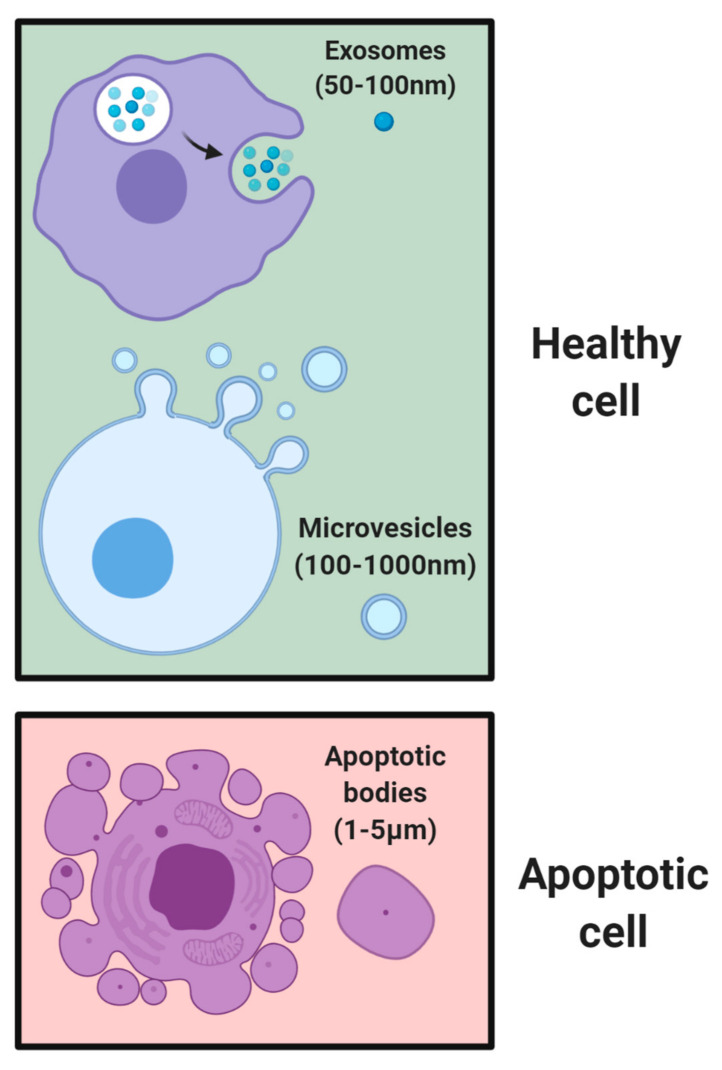
**Subsets of EVs based on size.** Healthy cells secrete exosomes of 50–100 nm in size and multivesicular bodies up to 1000 nm in size through blebbing of plasma membranes. Following cell death, apoptotic bodies of 1–5 μm in size are formed.

EVs are nano-sized proteo-lipid vesicles released by cells via exocytosis which are then taken up by recipient cells [1,2]. They play essential roles in normal homeostasis and disease initiation and progression [3]. Messages conveyed by EVs include signals for apoptosis [4], cell differentiation [5], survival [6], tissue repair [7], immune responses [8], and tumor growth [9]. EVs are composed of a complex bilayer membrane made of lipids, sugars, and proteins, permitting them to protect their cargo from degradation and transfer biological messages to other cells [10]. This cargo can be in the form of ribonucleic acid (RNA) [11], messenger RNA (mRNA) [12], micro RNA (miRNA) [13], proteins [14], or lipids [15]. Once EVs reach the target cells, they can deliver their biological message via receptor-ligand interactions, fusion with cellular membranes, or internalization via endocytosis or phagocytosis [16]. To date, three primary subsets of EVs have been characterized and distinguished based on their size (Figure 1). The smallest EVs are called exosomes, 50–100 nm in diameter, and primarily generated from multivesicular bodies and secreted by various cell types (e.g., immune, stem cell, and cancer) [17]. Micro vesicles range between 100–1000 nm and are secreted through blebbing of their plasma membranes [18]. Apoptotic bodies are micro-sized EVs (1–5 μm) normally secreted during apoptotic events [19,20]. It should be noted that there are a host of other subpopulations of EVs secreted by cells, with more being discovered in recent times [21,22]. One such example is exomeres, which were recently discovered as a nanoparticle with no biological function, but rich in metabolic enzymes and signature proteins [23]. However, this review will specifically focus on exosomes.

Inspired by the natural efficacy of EVs in cell-cell communication, synthetic nano-based approaches have been developed to mimic and/or amplify the messages delivered by EVs [24,25,26]. In this paper, we aim to review how leukocytes, erythrocytes, platelets, stem cells, and cancer cells communicate using EVs. We will review current research findings on how EVs from various cells have been used to target a variety of diseases. In addition, after explaining the limitations of current EVs, we will explore how these well-tuned communication methods can be engineered to further develop more efficient, standard, and innovative technologies to accelerate the use of EVs in the clinic.

## 2. Native EV Communication

**Figure 2:**
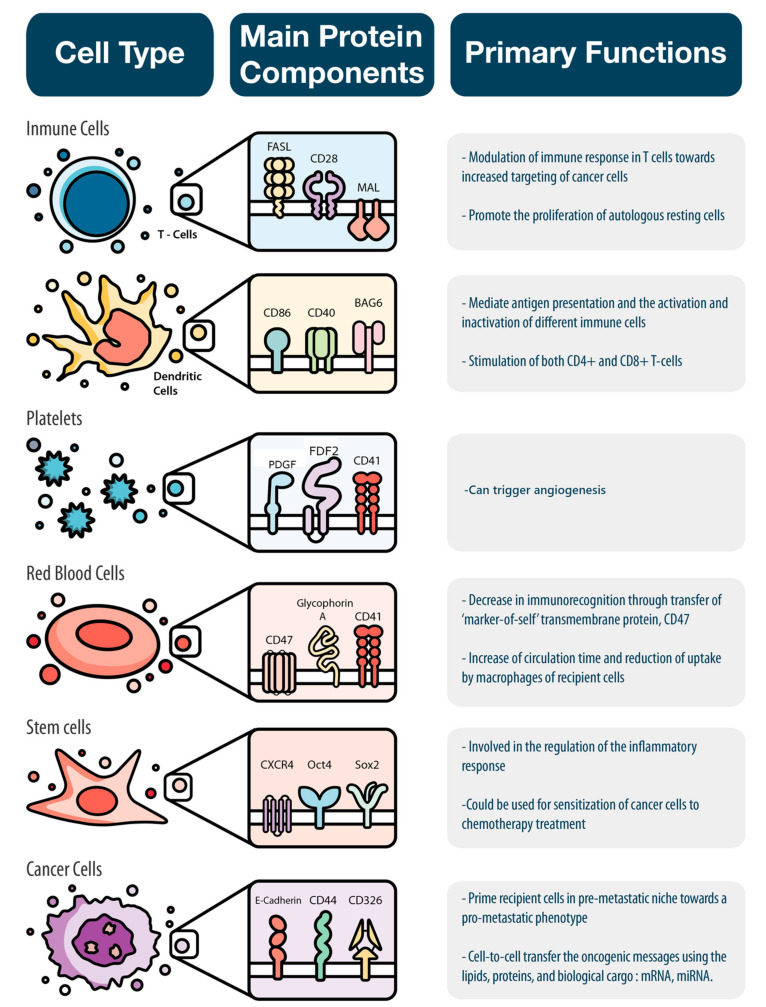
**EVs deliver specific biological messages according to the cell type from where they originated.** Immune cells, platelets, red blood cells, stem cells, and cancer cells were analyzed in this paper based on their main protein component and the specific biological message they convey.

There is an assortment of biological messages continuously sent between the various cells of our body to maintain physiological homeostasis. The content of the messages delivered by EVs is affected by the parent cell (i.e., sender), environmental cues (e.g., temperature and pH), and biological parameters (e.g., hormones and enzymes). We organized the following sections to describe how different cells use EVs to deliver specific biological messages (Figure 2).

### 2.1. Immune Cells

The immune system plays a critical role in the maintenance of homeostasis. The body’s immune response to foreign bodies is coordinated by many different cells: macrophages, T-cells, dendritic cells (DCs), and natural killer (NK) cells. These cells are endowed with different objectives and work together to defend our body against infectious organisms and other foreign invaders [27]. EVs released by immune cells serve as key mediators in cell-to-cell interactions. The secretion of these vesicles can be spontaneous or induced according to the cell type from which they originate [28]. For example, while T-cells and resting B-cells release EVs following the activation of cell surface receptors, DCs and macrophages constitutively secrete EVs [17]. Recent studies have demonstrated that T-cell based stimuli can also induce DCs to secrete EVs [29]. However, other work has found that the secretion of EVs by immune cells can be modulated by environmental factors such as receptor-ligand interactions and mitogenic lectins [30]. Nevertheless, the function of these secreted vesicles primarily depends on the cells from which they originate. The distinct structural and biochemical features of each EV type are directly tied to their unique function which can be either immune-activating [8] or immune-inhibitory [31]. The sections below summarize the features and functions of the various immune cell-derived EVs separated by the two arms of the immune system: innate and adaptive.

#### 2.1.1. Innate Immune System

Innate immunity is the body’s first line of defense upon pathogen detection. This arm of the immune system is nonspecific and comprises a general response to kill anything that is not identified as ‘self’. Primary mediators of this response include macrophages, DCs, and NK cells.

Macrophages are phagocytic cells that continuously circulate throughout the body in search of pathogens. Upon identification of one, macrophages phagocytose bacteria and viruses, process the pathogenic components, produce antigens recognized by effector immune cells, and secrete cytokines as signals to recruit other immune cells to the site of infection. As these cells process the foreign material, they also secrete EVs containing pathogen-associated molecular patterns (PAMPs) [32]. EVs further enhance the immune response by increasing cytokine production and mediating communication with effector T-cells. For example, it has been shown that EVs released by macrophages infected with *Salmonella enterica* and *Mycobaterium tuberculosis* carried PAMPs that induced cytokines production via activation of Toll-like receptors (TLRs). Another study demonstrated that exosomes from *M. tuberculosis-*infected macrophages carry major histocompatibility complex (MHC) class I and II costimulatory molecules capable of inducing memory in CD4+ and CD8+ T-cells.

In addition to having stimulatory effects on the immune system, macrophage EVs can also possess inhibitory effects. This behavior aligns with the double polarization profile of macrophages as either pro-inflammatory M1 or anti-inflammatory M2. In fact, analysis of miRNA content from activated macrophages has shown that EVs secreted from either M1 or M2 macrophages carry signatures of the corresponding phenotype. As a result, the behavior of these EVs in the disease context mimics that of the source cells. For example, M2-derived EVs promote migration of gastric cancer cells via activation of PI3K-Akt pathway, a behavior that is much like the migratory behavior induced by tumor-associated macrophages (TAMs) across many cancer types. Furthermore, these EVs possessed a molecular profile similar to the pro-tumor, immunosuppressive phenotype observed in TAMs, which includes increased expression of M2 markers (CD163 and CD206) and reduced expression of M1 markers (IRF5 and TNF-a). Additional examples, from a recently published paper by Biemmi et al. [33], describes a therapeutic approach to preserve the function of an ischemic heart by targeting circulating inflammatory EVs during the acute phase of myocardial infarction. This group of scientists tested the paracrine effect that is mediated by these EVs and how it induces direct cytotoxicity in cardiomyocytes. Moreover, when a chemical inhibitor of EVs biogenesis was injected after myocardial infraction the left ventricular ejection fraction was preserved in vivo.

Therefore, the molecular profile of macrophage-based EVs plays a key role in mediating the immune response within the disease context, where the content of these vesicles is vital to tipping the balance towards a pro- or anti-inflammatory response.

DCs are professional antigen-presenting cells (APCs) whose primary function is to capture, process, and present antigens to lymphocytes [34]. This process is vital to trigger the appropriate immune response and coordinate the cells to carry out the response. While DCs themselves act as direct communicators to effector immune cells, they also secrete EVs to improve this communication. In fact, DC-derived EVs have been shown to contain MHC class I and II molecules, costimulatory, and adhesion molecules that activate the T-cell response through both direct and indirect mechanisms. While antigen presentation from the EVs can directly activate T-cells, the EVs themselves can be internalized by other DCs that go on further to activate lymphocytes. Furthermore, the activation efficiency of these EVs directly correlates to the status of the DC source. Indeed, EVs derived from mature DCs induce higher T-cell activation than those isolated from immature DCs. This behavior has been studied in many different conditions, ranging from tissue regeneration [35] to cancer immunotherapy [36]. For example, DC-derived exosomes have been shown to specifically promote mesenchymal stem cell (MSC) migration without affecting their differentiation or proliferative potential. Probing the mechanism of this behavior, researchers discovered these EVs were enriched in chemo attractants (e.g., osteopontin and MCP-1) and metalloproteinases (e.g., MMP-2 and MMP-9). The induction of this migratory behavior can be exploited to increase MSC recruitment to injured sites and, thereby, further stimulate tissue regeneration. In addition, DC-derived exosomes have been heavily explored for their use in cancer immunotherapy as cell-free vaccines. Tumor peptide-loaded DC-derived exosomes were shown to completely eradicate tumors through activation of cytotoxic T-lymphocytes (CTLs). Furthermore, phase I clinical trials using these EVs have corroborated the safety and cytotoxic potential of this platform in melanoma patients. Using melanoma-associated antigen (MAGE)-loaded DC exosomes, researchers found that these EVs expressed NK lectin-like receptor ligand, significantly increased circulating NK cells, and induced IFN-y production. Taken together, these studies not only highlight the therapeutic potential of DC-derived exosomes, but also the crucial role they play in mediating communication across many cell types.

The primary role of NK cells in innate immunity is to destroy target cells through the release of cytotoxic molecules or the induction of apoptosis via activation receptor ligation [37]. EVs secreted by these cells express typical markers of NK cells, which include CD56, the apoptosis-inducing FAS ligand, and activating receptor natural killer group 2D (NKG2D). In addition, these EVs also carry cytotoxic molecules, which include perforin and granzymes. As one may expect, NK cell-derived exosomes have demonstrated significant cytotoxic activity against a variety of tumor cells, such as leukemia, melanoma, and breast cancer cells [38,39]. In vitro findings were further corroborated in an in vivo model of melanoma where tumor-bearing mice were treated with NK-derived EVs. Although the direct antitumor effect was mediated by the perforin and FAS ligand, intrinsic apoptosis pathways, such as caspase-3 and PARP, were also found to be activated because of the activity of NK EVs [40]. In addition, NK-derived EVs were found to downregulate the MAPK pathway, increase p38 tumor suppressor protein expression, and modulate TNF-a expression. These latter findings show that NK-cell-derived EVs not only exert cytotoxic effects on target cells, but also possess the ability to modulate the local tumor environment through inflammatory cytokines and tumor suppressor genes. This notion was further supported in a study using miR-168 loaded NK-derived exosomes against neuroblastoma [41]. EVs in this study not only exerted a cytotoxic effect against the tumor cells, but they also prevented TGFß-1 dependent immune evasion by the tumor cells. Although studies about the potential use of NK-cell-derived exosomes remain limited, experiments that have been performed demonstrate the potential these EVs possess in communicating and modulating the local immune microenvironment.

#### 2.1.2. Adaptive Immune System

The adaptive immune response can be viewed as a response that is molded and shaped over time as cells are exposed to antigens. This acquired immune response results in greater specificity of the immune response to a particular pathogen. Although components of the innate immune system mediate the activation of this response, the key effector cells involved are T- and B-cells.

The ability of T-cells to recognize a specific antigen induces a coordinated two-pronged response. The first involves activation of cytotoxic T-cells to destroy foreign material, while the second involves the priming of memory T-cells to recognize future exposures to the same antigen. T-cell derived EVs contain molecules found in other EV types, such as miRNA, perforins, and granzymes, and express common T-cell markers, including CD3, CD8, and TCR. Stemming from the stimulatory nature of T-cells, EVs secreted by these immune cells have been shown to promote the proliferation of autologous resting cells and promote tumor invasion to the lungs. Activated CD8+ T-cells were also found to inhibit tumor invasion by direct modulation of tumoral MSC via miR-298 [42]. In contrast, T-cell derived EVs can exhibit immunosuppressive properties. For example, activated T-cell derived EVs expressing CD95 induce apoptosis of bystander T-cells, which in turn triggers immune suppression and inhibits T-cell proliferation. These immunosuppressive properties have been proven useful in the context of graft-versus-host disease (GVHD), where overstimulation of the immune system results in chronic organ rejection. In fact, researchers were able to prevent GVHD following a kidney transplant by suppressing the immune response to the foreign organ through the systemic administration of isolated EVs following the transplant procedure. These studies highlight the ability of T-cell derived exosomes to act as both immune activators and regulators, much like the T-cells from which they are derived.

Key functions of B-cells include the production of antibodies, the presentation of antigens to T-cells, and the development of immune memory. The release of EVs from B-cells occurs in response to CD40 and IL-4 signaling. Isolated EVs show high expression of B-cell markers (i.e., CD19, CD45R), MHC I, MHC II, and tetraspanin proteins (CD81). Investigation of immune cell response to these B-cell derived EVs indicated significant increases in the number of NK, T-, and B-cells within the spleen five days postimmunization. Another study demonstrated that B-cell derived exosomes could not only activate allergen-specific T-cells, but also induced production of the anti-inflammatory cytokines IL-5 and IL-13 [43]. However, work focusing on B-cell exosomes has remained limited, and further studies are needed to fully elucidate the mechanisms by which this class of EVs acts and, thereby, unlock the therapeutic potential they possess.

### 2.2. Erythrocytes and Platelets

Erythrocytes, (i.e., red blood cells (RBCs)), and platelets are derived from a common progenitor cell. The primary role of RBCs is to transport oxygen from the lungs to the rest of the body and return carbon dioxide from the body back to the lungs for excretion. Platelets primarily serve to initiate the formation of clots in response to injury of blood vessels. In this section, we will focus on the crosstalk and interactions of EVs excreted by RBCs and platelets and the role that these EVs play in regulating homeostasis.

RBCs are the most abundant cell that circulates in the blood [44]. Due to their unique biconcave disk shape with a flat center, they are more flexible and have the ability to squeeze through blood vessels of varying sizes [45]. In addition, these cells circulate in the peripheral blood for an average of 120 days.

RBCs-derived EVs were discovered to exert a protective role on RBCs when in the presence of damaging molecules, such as oxidized proteins. This allowed RBCs to remain in circulation rather than being removed during the clearance of dangerous molecules. In addition, after being infected with *Plasmodium falciparum* (the parasite responsible for malaria), it was demonstrated that RBCs could release EVs, promote parasite survival in the host, and transmit to other mosquitoes. This EV-based mechanism of communication represents a possible target for the future development of agents capable of blocking the transmission of this parasite. By preventing the release of EVs, it is possible to inhibit the survival of the parasite in the RBCs and prevent transmission to other mosquitos.

Platelets are cells that circulate in the blood and play a key role in maintaining homeostasis and blood vessel integrity. When homeostasis is disrupted and bleeding occurs, platelets adhere to the injured blood vessel and initiate the blood coagulation process. Currently, there is a strong interest in the isolation of platelet-derived EVs and their role as functional mediators of the clotting cascade. For example, platelet-derived EVs were found to act as angiogenetic boosters after vascular injury [46]. Furthermore, scientists found that platelet-derived EVs are filled with growth factors such as vascular endothelial growth factor (VEGF), basic fibroblastic growth factor (FGF2), and platelet-derived growth factor (PDGF), which assist in vascular regeneration. This was further demonstrated in EVs that triggered angiogenesis following injury due to a stroke [47].

### 2.3. Stem Cells

Stem cells (SCs) are a small population of cells involved in the homeostasis of cells and tissue. Generally speaking, SCs can be divided into two major categories: (1) embryonic stem cells (ESCs) and (2) adult stem cells (ASCs). Both ESCs and ASCs are characterized by unlimited proliferation and self-renewal capabilities. However, while ESCs present pluripotency capacity and can differentiate into the three embryonic germ layers (mesoderm, ectoderm, and endoderm), ASCs can give rise only to the cell subtypes of the specific tissue where they reside. Based on these important differences, the applications for EVs from ESCs and ASCs vary.

#### 2.3.1. Embryonic Stem Cells

Due to their unlimited proliferation potential and differentiation ability, ESCs have extensively been explored as a viable tool to repair damaged or diseased tissues. In particular, ESCs have been applied to transplantation, regenerative medicine, myocardial infarction and ischemia [48], and wound healing after surgery. However, the current use of ESCs for cell-based therapies is a matter of debate due to ethical concerns and the high risk of malignant cell transformation (i.e., ectopic tumor formation). Indeed, as a result of their incomplete differentiation status, ESCs could potentially lead to the formation of teratomas. On the other hand, EVs released by ESCs are considered an important source of bioactive molecules endowed with the ability to modulate their physiologic environment. Thus, EVs from ESCs could represent a novel cell-free solution associated with a reduced risk of immune reaction and tumor induction.

Several studies have confirmed the role of EVs in mediating the communication between ESCs and other cell types (e.g., Mueller retinal cells and embryonic fibroblasts) [5,49,50,51]. After internalization by target cells, EVs released by ESCs induced the expression of stem cell-specific markers (e.g., Scl, HoxB4, GATA2, and Nanog), growth factors, and mRNAs. This increased expression leads to phosphorylation of signal transduction mediators such as MAPK p42/44 [52] that facilitate the reprogramming of target cells [53,54]. In summary, these results indicate that ESC-derived EVs are able to transfer SC-associated features (e.g., self-renewal and pluripotency) to differentiated cells, thus inducing their de-differentiation in order to repair injured tissues. In addition, EVs from ESCs can stimulate the rapid expansion of other ESCs and increase their self-renewal pluripotency. The unlimited abilities to proliferate and differentiate offered by ESCs-derived EVs could pave the way for future studies that aim to exploit the reparative functions of these biological messages.

#### 2.3.2. Adult Stem Cells

Adult stem cells are a rare cell population in the human body that can repair or replace injured or diseased tissue. Although perceived to have less risk compared to ESCs, ASCs still present technical complications linked to their isolation, culture-induced senescence, immune-mediated reaction, and genetic instability.

One of the more studied and applied subsets of ASCs are mesenchymal stem cells (MSCs). MSCs originate from the mesodermal germ layer, but are commonly found in bone marrow, adipose tissue, and the umbilical cord, MSCs are endowed with multipotent potential as they can differentiate, under specific cytokine-mediated stimuli, into osteoblasts, adipocytes, and chondroblasts. In addition, MSCs play a key role in cancer progression and have shown promise in targeting inflammation. On this note, the tumor microenvironment secretes cytokines (e.g., SDF-1, EGF, PDGF, TNF-α and IL-8) that recruit MSCs into the tumor mass. Once in the tumor, MSCs can interact with surrounding cells by (1) modulating gap junctions; (2) enhance the formation of nanotubes (e.g., protrusions that extend from the plasma membrane of cells); or (3) indirectly releasing cytokines and EVs [55]. Furthermore, MSCs-derived EVs exert pro-tumorigenic effects by enhancing angiogenesis, drug resistance, immune escape, and metastatic progression to other organs [56,57]. This tumorigenic enhancement was mediated by the lipid composition of EVs enriched in molecules such as diacylglycerol, sphingomyelin, and ceramide. These molecules are involved in the regulation of proliferation, apoptosis, migration, and tumorigenesis [58,59]. While the effects of EVs derived from MSCs might be linked to the recipient cells, as indicated by tumor type and stage [60,61], the cell source from which the EVs originated also plays an important role. For example, it was demonstrated that EVs isolated from adipose tissue-derived MSCs stimulated tumor growth and proliferation. Conversely, EVs produced by MSCs isolated from the bone marrow and umbilical cord inhibited tumor proliferation and induced apoptosis [62]. This demonstrates the importance of the tissue source for MSC-derived EVs as different origins can have vastly different downstream effects.

In terms of regenerative medicine, MSC-derived EVs have shown promise for the treatment of wounds [63], tissue repair via matrix remodeling and inhibition of the epithelial to mesenchymal transition [64], and bone regeneration. It was discovered that EVs released from MSCs accelerated re-epithelialization, reduced scar widths, stimulated angiogenesis, and promoted collagen synthesis at the wound site [65]. In conclusion, all SCs possess three specific properties: (1) the ability to divide and self-renew, (2) to give rise to specialized cell types, and (3) to be unspecialized. These properties are also shared with SC-specific EVs, which hold considerable promise for tissue regeneration and provide insight into how these properties can be hijacked for a therapeutic benefit.

### 2.4. Cancer Cells

During cancer progression, tumor cells acquire traits that allow them to stimulate their own growth, evade apoptosis and the immune system, sustain angiogenesis, invade local tissues, and metastasize to distant organs. Tumor-derived EVs affect several of these processes, conferring to tumor cells aggressive phenotypes (i.e., increased invasion, promoted metastasis formation, amplified drug resistance), and influencing the tumor microenvironment [66,67,68,69]. Moreover, tumor-derived EVs transfer their oncogenic messages using lipids, proteins, and biological cargo that are embedded within their membrane. The interior cargo of tumor-derived EVs alters the characteristics, functions, and phenotype of the receiving cells. For example, researchers discovered that tumor-derived EVs from melanoma and pancreatic cancers have the ability to prime target cells in the pre-metastatic niche (e.g., bone, lung, liver) towards a more pro-metastatic phenotype that favors metastatic development [70,71].

The initial idea of using tumor EVs for drug delivery emerged following the discovery of their cell-specific tropism. The tropism of tumor cell EVs is due to the expression of specific surface proteins, such as the tetraspanins, growth factor receptors, and adhesion molecules [72], which are inherited from donor cancer cells [73,74]. However, the specificity of targeting remains highly debated [75]. Some studies have demonstrated successful specific targeting. For example, EVs from brain tumor cells with high expression of CD63 tetraspanins demonstrated the ability to successfully cross the blood–brain barrier, permitting the delivery of doxorubicin to tumors in the brains of zebrafish [76]. In addition, EVs derived from lymphoma cells encapsulating curcumin, an anti-inflammatory agent, provided effective treatment by decreasing inflammation and regulating tumor propagation in mice with brain tumors, as well as in mice with endotoxin-induced septic shock [77,78]. However, there is concern surrounding the use of tetraspanins due to their role in mediating tumor growth. In addition, it is worth noting that using tumor samples of human origin could be the source of EVs tropism rather than the actual homing of EVs to tumors.

Rather than capitalizing on the specificity of native surface proteins on EVs for targeted delivery, an alternative strategy is to load cells before EV isolation and use the native packaging mechanisms in donor cells to create drug-loaded EVs without post-secretion manipulation. Scientists demonstrated this technique by collecting tumor-derived EVs from various tumor cells (e.g., hepatocellular, lung, melanoma, lymphoma) that were initially incubated with chemotherapeutic drugs (e.g., doxorubicin, methotrexate, cisplatin, camptothecin), and then irradiated with UV light to induce apoptosis and stimulate the formation of drug-loaded EVs [79]. These tumor-derived, pre-loaded EVs were demonstrated to be used as an effective therapy for liver and ovarian cancer with reduced adverse effects. The findings from this study prompted the initiation of clinical trials to evaluate the effect of tumor-derived pre-loaded EVs on malignant ascites and pleural effusions [80]. However, despite the excitement surrounding tumor-cell-derived EVs, the use of tumor EVs in the clinic is less enthusiastic due to the potential risk of exacerbating the patient’s condition due to the role of EVs in communicating cancer growth, progression, invasion, and survival.

In cancer patients, EVs were found in the spleen and lymph nodes of cancer patients with lymphoma. This led to the discovery that the spontaneous shedding of EVs from highly metastatic cancer cells could transfer their metastatic ability to poorly metastatic cancer cells [81], which could potentially spread cancer to organs that would have otherwise remained cancer-free. Furthermore, additional studies revealed that EVs secreted from tumors contained distinct integrin expression patterns that directed their tropism to preferentially fuse with cells to prepare the pre-metastatic niche [70]. These tumor-derived EVs transferred this oncogenic message via plasma membrane components originally derived from the highly metastatic cancer cells. Furthermore, reports revealed that EVs released from glioblastoma tumor cells, enriched in mRNA, miRNA, and angiogenic proteins, were internalized by nearby normal microvascular endothelial cells in the brain. This resulted in increased angiogenesis and tumor progression [82]. Although there are potential therapeutic applications for tumor-derived EVs, several concerns remain about their therapeutic use due to their ability to communicate with surrounding recipient cells within the tumor microenvironment and deliver biological and genetic information that aids in the growth and progression of tumors.

Other strategies for cancer immunotherapy include activating or stimulating immune responses by activating T-cells to a broad range of tumor antigens. As previously discussed, mature DCs can activate immune responses via T-cells but are limited by the poor antigen uptake and cross-presentation on MHC class I molecules. Interestingly, ascites recovered from cancer patients were discovered to have high levels of tumor-derived EVs endowed with tumor antigens, including MHC I molecules [83,84]. These EVs could be used as a source of antigens to transfer tumor antigens to DCs, thus enlarging the cross-presentation process for effective cancer immunotherapy. Investigators tested the ability to utilize EVs derived by MHC molecules on DCs and were able to demonstrate that DCs exposed to tumor-derived EVs were internalized and cross-presented to MHC class I molecules. This subsequently triggered activation of the MHC class I T-cell clones, increased the number of CD8+ T cells, and stimulated the release of interferon-gamma. This suggests that DC EVs hold promise for a potential immunotherapy treatment for cancer. Additional animal experiments in mice showed that DCs loaded with tumor-derived EVs induced effective tumor rejection and could potentially be used in conjunction with immune checkpoint inhibitors (i.e., specific molecules that may initiate an immune response). These results led to the development of clinical trials to investigate the use of tumor EVs for the treatment of post-resection glioma patients. However, recent findings suggested tumor EVs may suppress immune responses, and thus more attention needs to be given to the state of the immune system of donor cells in future experiments [80,85].

## 3. Engineered EVs as Drug Delivery Vehicles

**Figure 3:**
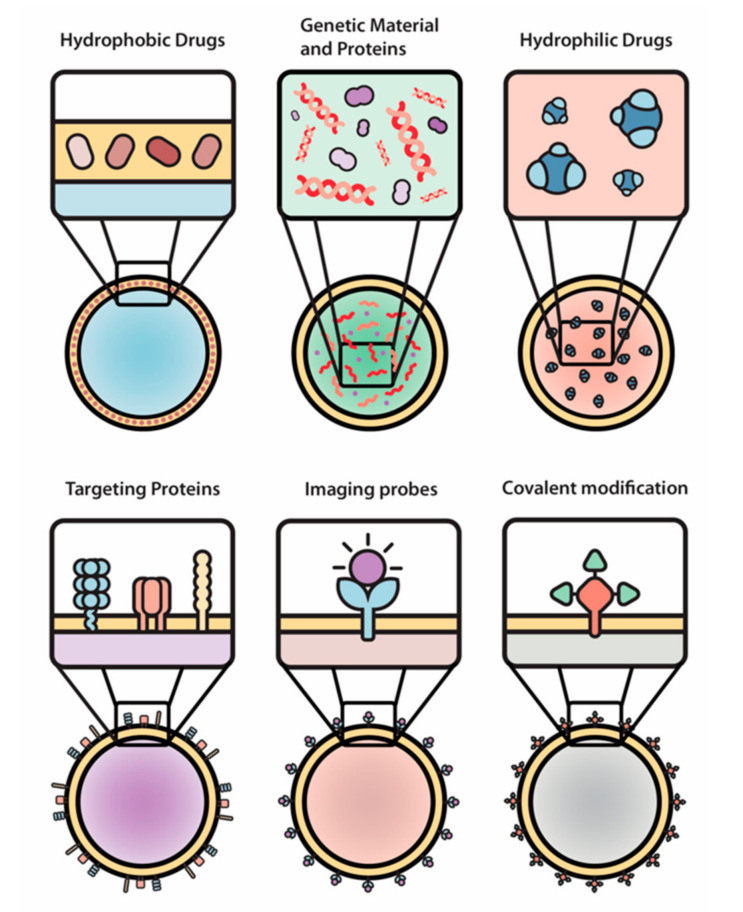
**The engineering of EVs allows for a wide array of modifications of therapeutic payloads and surface moieties.** Hydrophobic drugs, genetic material and proteins, hydrophilic drugs, targeting proteins, imaging probes, and covalent modifications were used as examples in this paper.

In general, drug delivery system developers are seeking ways to optimize nanocarriers’ properties. More specifically, they are trying to improve circulation time, prevent accumulation in filtering organs, and overcome native biological barriers (e.g., blood-brain, endo-lysosomal, vascular, etc.) that prevent specific and directed targeting [86]. While several of the previously mentioned optimization methods have been explored [87], further improvements are still necessary. EVs, as natural nanocarriers, have been modified to carry both natural and synthetics small molecules (e.g., curcumin, ponatinib, and doxorubicin) [88,89,90], therapeutic proteins (e.g., catalase) [91,92], or genetic cargo (e.g., two different siRNAs) [93], demonstrating their versatility for drug delivery (Figure 3).

The main methods to develop EVs for clinical use include (1) the modification of donor cells before EVs secretion and (2) surface engineering of natural EVs after secretion by donor cells [94]. Following the first method, the donor cells are often exposed to various stimuli in order to modify both the payload and membranes’ components of the EVs [95]. For example, DC-derived exosomes were engineered to express a modified muscle and brain tissue targeting marker, Lamp2b, through transfection of the donor DCs with engineered plasmids [96]. Successful transfection of the source cells resulted in the generation of a sustained pool of EVs expressing this specific marker. This methodology has served as the basis of companies, such as Evox Therapeutics, which are working to engineer native EVs [97]. Even the culture conditions of the source cells have been shown to improve EV output. For example, 3D cultures of MSCs yielded over 20-fold more EVs when compared to EVs obtained from 2D culture [98]. Furthermore, EVs derived by this method were also shown to demonstrate more biological activity due to the enrichment of key proteins needed for the efficient delivery of siRNA to neurons in vitro. Instead, the second method exploits different ways to modify EVs’ surface markers after isolation from the donor cells. This is most commonly done through chemical means, such as utilizing click chemistry for the conjugation of ligands [99]. This strategy was tested by Myung Soo et al. and is shown to improve paclitaxel encapsulated exosomes’ targeting lung cancer cells by incorporating aminoethylanisamide-polyethylene glycol to their surface. Another example of this chemical modification is the incorporation of aminoethylanisamide-polyethylene glycol on macrophage-derived exosomes for the improved targeting of overexpressed sigma receptors on lung cancer cells [100].

Nano-engineers have also developed different strategies to alter EVs’ payload after cellular secretion of EVs. This cargo alteration can be done using two different methods: passive and active EVs loading. Passive loading does not involve energy investment or any other external component besides the EVs and the cargo that is to be loaded. Hydrophilic and neutrally charged molecules are able to diffuse through the bilayer membrane based on concentration gradients, while hydrophobic components can integrate within the hydrophobic membrane due to hydrophobic or electrostatic interactions. Zhuang et al. have demonstrated this kind of passive encapsulation using curcumin or JSI124 (cucurbitacin I) into EL-4 exosomes by incubating at 22 °C for 5 min [78]. Active loading involves the input of external energy to disrupt the integrity of the hydrophobic membrane bilayer of EVs. In this way, the selected cargo can either diffuse into the hydrophilic core or gets trapped within the EVs membrane. Currently, several approaches such as electroporation, freeze and thaw cycles, and phosphate gradients have been used to disrupt the membrane bilayer in order to achieve active encapsulation and permit higher cargo loading efficiencies. Due to the structural similarity of liposomes and EVs, the methods used for loading liposomes can also be utilized for EVs. For example, the high shear stress used to actively load collagenase proteins into the core of liposome nanoparticles using the extrusion method can be transferred to the loading of EVs. Other techniques used include sonication that interferes with the membranes of EVs by inducing acoustic waves and electroporation that interferes with the membranes of EVs by inducing electric fields.

## 4. New Approaches to Engineer EVs

While the engineering of native EVs themselves represents one approach to further modify these vesicles, another strategy involves combining the biological features of EVs with the tunable properties of synthetic NPs. For example, the development of synthetic liposomal nanoparticles (NPs) using either lipids or proteins found in native EVs has been shown to enhance the properties of natural EVs by mimicking their native features [101,102]. One reported approach describes the fusion of 1,2-dioleoyl-sn-glycero-3-phosphocholine (DOPC) and 1,2-dioleoyl-sn-glycero-3-phospho-L-serine (DOPS) (i.e., synthetic lipids) with raw 264.7 (murine macrophage cells) cell-derived EVs in order to control the new NP’s surface charge, fusogenic properties, and colloidal stability [103]. This new approach aims to transfer the past knowledge acquired from synthetic NP formulations, such as stability and scalability, to optimize the process of NPs’ synthesis and ensure their facile translation for clinical use. The following sections describe how unique and specific biological properties of immune cells, platelets, red blood cells, stem cells, and cancer cell EVs can be transferred to NP systems to mimic native EVs [104,105,106].

### 4.1. Immune Cells

**Figure 4:**
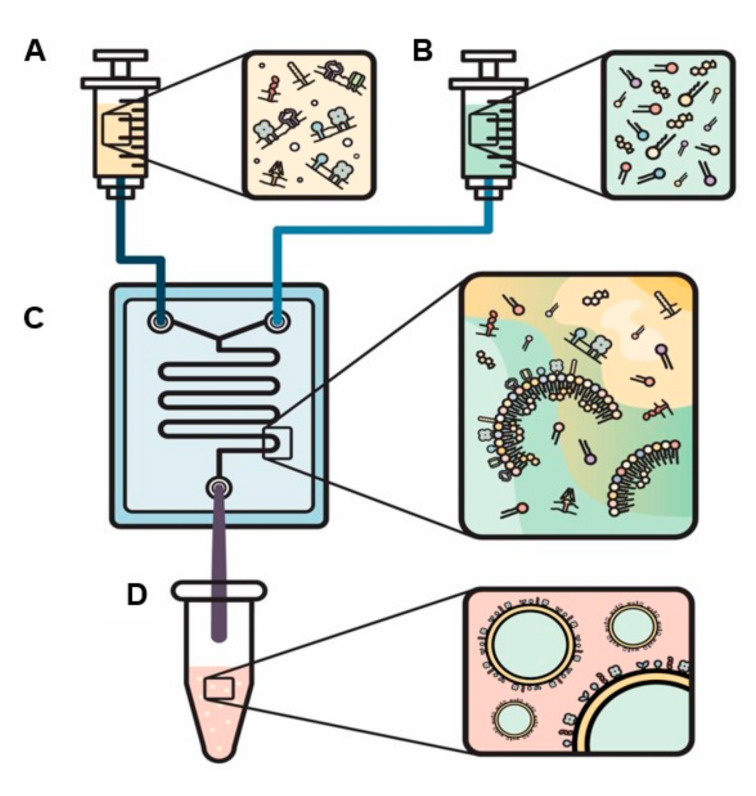
**A novel method to actively load proteins into the bilayer of engineered EVs.** Solutions are loaded into two separate syringes: (1) proteins within the aqua phase (**A**) and (2) lipids and cholesterol within the organic phase (**B**). The syringes are injected into a chip (**C**) and specific ratios of organic to aqua phases and flow ratio (the speed that the fluids expelled from each syringe mix) are selected to produce biomimetic EVs (**D**).

Novel engineered EVs with leukocyte membrane proteins were developed in order to combine leukocytes’ biological targeting properties with the nanoparticles’ ability to deliver different types of cargo. The specific biological properties of leukocytes that can be exploited include evasion from the hosts’ immune system, the ability to cross biological barriers, and specific targeting of tissues via their cellular membrane interactions. This allows particles to avoid opsonization from the immune system, reduce sequestration in filtering organs, communicate with endothelial cells through receptor–ligand interactions, and transport a payload across an inflamed reconstructed endothelium [85,107,108,109]. Recent efforts have demonstrated similar outcomes using liposomal-like vesicles fabricated from leukocyte-derived membranes–“leukosomes”. Leukosomes are made of a liposomal lipid backbone with membrane proteins derived from leukocytes (Figure 4) [110]. Utilizing leukosomes was found to achieve an enhanced affinity towards activated endothelium, making this specific type of EVs promising as a theranostic tool [105]. Recently, Boada et al. demonstrated that by encapsulating Rapamycin inside leukosomes, they have managed to both target inflamed endothelia, which reduces this target drug’s effects, and reduce vascular inflammation by almost 2-fold compared to non-treated and rapamycin-treated mice. In this, they managed to slow the progression of the murine model of atherosclerosis [111].

In addition to the use of whole leukocyte populations for novel engineered EVs, researchers have explored the use of specific immune cell membranes for a variety of applications. Given their key roles in mediating and executing the immune response in different disease conditions, T-cells and macrophages have been commonly used immune cell sources for these EVs. For example, CD4+ T-cell membrane coated polymeric nanoparticles were developed to target HIV viral particles and were shown to act as decoys, preventing the virus from attacking the intended host targets. In the context of sepsis, macrophage-coated nanoparticles have been utilized for endotoxin neutralization and the sequestration of inflammatory genes. These engineered EVs demonstrated their ability to directly modulate the immune response involved with the disease. In the context of cancer, immune-cell-based engineered EVs have been used as both drug delivery systems and agents for photothermal therapy. More specifically, macrophage membrane-derived vesicles were coated onto Magnetite (Fe_3_O_4_) NPs. ‘NKsomes’, which consisted of doxorubicin-loaded NK cell membrane coated nanoparticles, exhibiting superior affinity to tumor cells while maintaining a circulation time of 18h in the blood [112]. In contrast to the classical drug delivery approach often used to target the tumor with chemotherapy drugs, macrophage-based nanoparticles have been used as phototherapy agents that locally heat the tumor upon activation from an external light source. Gold nano shells coated with the membranes of macrophages were shown to achieve 4-fold longer circulation time than bare NPs alone. Furthermore, the macrophage membrane protein coating also enhanced the tumor accumulation, due to longer systemic delivery via the enhanced permeability and retention effect. These examples of immune cell engineered EVs highlight the versatility these technologies offer in both targeting and treating the disease by mimicking components of the body’s own defense mechanism.

### 4.2. Erythrocytes and Platelets

Long-circulating delivery systems are important because they increase the probability that engineered EVs accumulate at the desired target site and decrease the need for repeated treatments. These factors help to achieve the intended therapeutic effects [113]. Due to these favorable features, as RBCs extrinsically exhibit long circulation times, scientists have exploited the membranes of RBCs in order to evade the immune system and allow the EVs to reach the target site [104]. Moreover, scientists have exploited RBCs membrane proteins, size, shape, and morphology to engineer EVs with superior circulation times [114]. These RBCs EVs have been used for the delivery of RNA drugs including antisense oligonucleotides, Cas9 mRNA, and guide RNAs with no observable cytotoxicity [115]. A previous study used membranes from RBCs to cloak poly lactic-co-glycolic acid (PLGA) NPs. Using this technique, the particles were able to lower immune recognition [104]. Specifically, functionalization of PLGA biomimetic EVs with freshly isolated RBC membranes allowed the surface of PLGA nanoparticles to closely mimic the protein and surface composition of RBCs. In addition, further characterization revealed that grafting of the membrane proteins successfully transferred the ‘marker-of-self’ (i.e., the transmembrane protein CD47) to EVs. The incorporation of CD47 on the EVs enabled prolonged circulation in the blood (11% retention versus 2% retention at 48 hours) [116].

During vascular injury, platelets adhere to the vascular wall and target sites of injury to promote hemostasis. This ability to recognize the site of injury is a promising approach that has been utilized to target injury sites and promote healing. To exploit this property, platelet-like EVs were based on a removable polystyrene core, which is coated with protein moieties found on platelets (e.g., von Willebrand factor) [117]. Critical surface ligands that bind to activated platelets (e.g., fibrinogen-mimetic peptide) were incorporated onto the membrane, creating EVs that mimic the shape and flexibility of platelets. In addition, platelet-membrane coated particles were found to significantly reduce internalization by macrophage-like cells, demonstrated a preferential binding to damaged arteries, and provided a ~65% reduction in bleeding in a mouse model. This allowed EVs to escape detection by the immune system and target only the vessels that were damaged. Inspired by these physical parameters, scientists have engineered platelet-like biomimetic EVs for targeting vascular injuries by mimicking platelet shape, flexibility, and complex surface interactions.

### 4.3. Stem Cells

Novel engineered EVs with membrane proteins from stem cells were developed in order to regulate the inflammatory response and to explore their potential use as an anti-tumor therapy. The majority of attempts to engineer these stem cell EVs have been done in vitro by inducing genetic modifications on the parent cells (e.g., MSC). These modifications produced stem cell EVs that demonstrated superior therapeutic efficacy.

MSCs can be engineered in vitro by stimulation with different cytokines (e.g., interferon gamma and tumor necrosis factor alpha) in order to increase the expression of programmed death-ligand 1 (PDL-1) and MHC class II molecules on secreted EVs. These secreted EVs can then be used for applications where regulation of the inflammatory response is needed [118]. For example, stem cell EVs that overexpress TNF-related apoptosis-inducing ligand have been shown to induce apoptosis in lung, breast, and renal cancers [119]. This shows great promise for utilizing stem cell EVs to trigger directed apoptosis only in cancer cells. In addition, biomimetic engineered EVs produced from the membranes of MSC (e.g., “Nano-ghosts”) have also shown promise as a form of gene therapy to target tumors. This targeting was tested on PC3 (prostate cancer) and MCF7 (breast cancer) positive tumors.

### 4.4. Cancer Cells

The natural tropism of cancer cell-derived EVs for tumors, the pre-metastatic niche, and their potential use for immunotherapy inspired researchers to create engineered tumor EVs. As a proof-of-concept, researchers demonstrated that engineered EVs created by coating PLGA nanoparticles with membranes from MDA-MB-435 and B16-F10 cells exhibited a successful in vitro immune response in T-cells and targeting of cancer cells. For immunotherapy, researchers used engineered EVs from B16-F10 incorporated with monophosphosphoryl lipid A and showed promising in vitro results with a significant upregulation of DCs maturation markers that led to the stimulation of T-cells. For drug delivery applications, engineered cancer EVs took advantage of the inherent homotypic binding observed in cancer cells [120]. Engineered EVs from MDA-MB-435 exhibited a 40-fold increase in uptake in cancer cells. Based on these experiments, it was demonstrated that engineered cancer EVs could be used to successfully deliver tumor antigens to DCs and increase the affinity of EVs to target cancer cells for future applications in vaccine and drug delivery.

The possibility of developing EVs that have the ability to target cancer cells spurred the development of additional engineered cancer EVs for cancer therapy. Different cancer EVs were created by following a similar top-down approach to coat nanoparticles with cancer cell membranes. By taking advantage of the homotypic binding of cancer cells, investigators tested the ability of 4T1 (murine breast cancer cells) to engineer cancer-engineered EVs for in vivo therapy [121,122]. In these studies, investigators confirmed that engineered cancer EVs successfully targeted 4T1 primary and metastatic tumors and avoided rapid clearance by the mononuclear phagocyte system due to homotypic binding and the expression of CD47 on cancer cells, a protein critical for suppressing uptake by macrophage cells [123].

Effective tumor therapy was also achieved using engineered cancer EVs that were loaded with chemotherapy in order to overcome the drug resistance of tumor repopulating cells [124]. Using this method, scientists discovered the delivery of paclitaxel increased by 4.3- and 3.7-fold in primary tumors and distant lung metastasis, respectively when compared to Taxol, an FDA-approved version of paclitaxel. Treatment in mouse models revealed that engineered cancer EVs had a significant improvement in growth inhibition of both primary and metastatic tumors compared to Taxol. In addition, by switching out the polymeric core used to create engineered cancer EVs, investigators demonstrated the ability to use laser triggered production of heat or reactive oxygen species [122] for cancer therapy. 4T1-coated gold nanocages and 4T1-coated porphyrinic metal-organic nanoparticles were engineered to enable hyperthermia-triggered release of doxorubicin and generate reactive oxygen species using photodynamic therapy, respectively. Using this approach, engineered cancer EVs retained selective targeting to 4T1 tumors and were effective at inhibiting the growth of primary and distal (lung and liver) metastasis. Furthermore, through blood biochemistry tests and histopathological analysis, the engineered cancer EVs were confirmed to exhibit good biocompatibility in vivo.

Engineered cancer EVs were also created to elicit antitumor immunity for vaccine development. These EVs were composed of PLGA nanoparticles loaded with CpG oligodeoxynucleotide 1826, a potent immunological adjuvant, and coated with cell membranes from murine B16-F10 melanoma cells. These engineered melanoma cancer cell EVs exhibited potent DCs maturation in vitro and in vivo that resulted in a significant generation of native T cells. Furthermore, these engineered cancer EVs demonstrated effectiveness in the prophylactic setting. Vaccination with engineered cancer EVs showed that 86% of mice had no tumor occurrence 150 days after tumor cell challenge, whereas controls had a median survival of only 20 days. However, the investigators discovered that engineered cancer EVs alone were not sufficient for treating tumor cells previously implanted. To achieve adequate efficacy, it was determined that combination therapy of engineered cancer EVs with a cocktail of checkpoint inhibitors was required. Using this combination strategy, engineered cancer EVs plus a checkpoint inhibitor, provided a significant survival advantage, extending the median survival by 11 days compared to either engineered cancer EVs or the checkpoint inhibitor cocktail alone. This demonstrated that engineered cancer EVs were able to act synergistically with anticancer therapies, hyperthermia, photodynamic therapy, and immunotherapies to modulate the tumor microenvironment and immunity to provide elegant solutions for cancer therapy, imaging, and vaccine development.

## 5. Further Consideration for the Translation of EVs

In this review, we highlighted the biological importance of paracrine cell-to-cell communication and elaborated on how different cells can send specific messages to communicate and regulate physiological of pathological events. In addition, we described different methods to engineer EVs to enhance their performance and use them for therapeutic applications.

Although EV-based treatments hold great therapeutic potential, several substantial hurdles remain to be addressed before utilizing EVs in clinical practice. Standard operating procedures (SOPs) for their isolation [125], characterization [126], and storage [127] have yet to be established. Moreover, current SOPs for EVs characterization vary according to the isolation technique used, as well as the origin cells. Hence, the development of consistent SOPs to characterize the physical features of EVs is crucial for any future translational application into the clinic.

To date, several standard procedures have been utilized for the isolation and characterization of EVs. Researchers have divided the isolation techniques for EVs into three main classes: ultracentrifugation, adsorption to magnetic/non-magnetic beads, and size exclusion chromatography [128]. In addition, three methods have been predominately used for the high throughput characterization of EVs: a nanoparticle tracking system, tunable resistive pulse sensing, and high-resolution flow cytometry. Unfortunately, these three technologies result in data inconsistency. Furthermore, without strong technical knowledge and awareness of the instrument settings (e.g., detection threshold, camera level, and dilution factor), false measurements are likely to occur [126].

Adequate storage methods for EVs have also not been established. Nevertheless, storage methods are urgently needed for the clinical use of EVs as some conditions can alter both the physical and biological properties of EVs. For example, some studies reported that reducing the temperature for antibacterial purposes can directly affect the size and the number of EVs, while the addition of cryoprotectants will result in the partial lysis of EVs [129,130]. The temperature of storage is another highly debated topic. Some studies claimed that −80 °C is a reliable condition because it has been demonstrated that storage at this temperature conserved EVs, while −20 °C resulted in the loss of a significant percentage of EVs. On the other hand, other researchers showed that EVs should be used as soon as possible after their extraction to avoid their disruption. Though the debate is still ongoing, a quick usage following synthesis seems to be the most common procedure with regard to the storage of isolated EVs [127].

## 6. Conclusions

There are many open questions and challenges that need to be addressed before any substantial progress can be made in order to use EVs in clinical practice. Ongoing research has focused on developing the best EV platform for the target application by mimicking either the EV’s surface or morphology. To evade the immune system, leukocytes-mimetic have been developed by integrating leukocytes membrane protein into the EVs’ lipid bilayer. To achieve higher circulation time, RBCs and platelet EVs have been fabricated by either (a) engineering the EVs’ surface with RBCs and platelet membrane proteins or (b) by mimicking the unique morphology of either of these cells. MSCs EVs have been selected to decrease inflammation and tissue regeneration. Notably, these EV surfaces were not only engineered by integrating MSCs membrane proteins but also their membrane lipids. Cancer cell EVs have been used in order to specifically target cancer infected cells by integrating the cancer cells’ membrane proteins to the EVs’ surface. There is no rule of thumb to define EVs that best mimics the true biological state. Therefore, each engineered EV should be designed for its future biological task. Fabrication challenges include: (1) the amount of proteins on each EV membrane; (2) the ratio between the proteins to the EV backbone (i.e., lipids, polymers, etc.) on each EV; and (3) the number of injected EVs. All these properties should be taken into consideration when designing EVs. Moreover, scientists must determine how to best define and measure the physiological properties of EVs. By defining these properties, it would then be possible to determine which EVs are ready to be translated for efficient therapy for humans. Finally, in order for EVs to truly be used in patients, large-scale manufacturing procedures that do not interfere with the therapeutic potential of EVs must be developed. Without the ability to scale-up the engineering of EVs, it will be prohibitively expensive to use them in clinical practice.

Overall, while nanomedicine has showcased substantial promise in the delivery of therapeutics, a new generation of delivery systems is emerging stemming from biomimetic-based approaches. Using the body’s own tools to communicate, coupled with engineered upgrades (e.g., imaging probes, genetic cargos, and specific membrane proteins), EVs have the potential to provide significant advantages over current synthetic drug delivery systems. These systems will help overcome major challenges faced by native EVs, including scalability, reproducibility, and limited cargo encapsulation. Although each biomimetic EV will demand additional studies to address stability, toxicity, biodistribution, pharmacokinetics, and efficacy, these engineered systems have been shown to mimic native EVs by transferring specific biological markers to synthetic delivery systems or by mimicking natural EVs in shape and size. Utilizing the synergistic effect of the engineered systems to convey specific biological messages holds tremendous promise and potential to improve future therapeutic outcomes.

Future studies should focus on EVs engineering and will require the complex molecular composition of these structures for specific therapeutic applications. However, such strategies require the development of novel biotechnological techniques allowing for the precise tuning and characterization of EVs to specific cell types or tissues. In addition, determining what cargo needs to be loaded into EVs to optimize specific and direct biological pathways is necessary. To advance the current engineering of EVs, new techniques are urgently needed that remove unnecessary components and, at the same time, isolate specific proteins. With these tools in hand, EVs can be manufactured without compromising their overall function.

Nevertheless, considerable work is currently ongoing to decipher cell–cell communication in an effort to develop novel therapies for a variety of diseases. The unique biological message encrypted within the EV’s “cloak” (i.e., surface) could hold the promise of gaining insight into how to better recognize, interact, communicate, and regulate various cellular functions. By transferring these properties to engineered EVs, we could achieve superior success by utilizing and capitalizing on the body’s natural properties.

This work has categorized EVs by the origin of specific cell carriers, described their physiological role, and summarized the updated literature surrounding this topic. It also discussed the main challenges and roadblocks that prevent EVs from being used in clinical practice. Finally, this paper reviews novel methods to synthetically fabricate EVs while still emphasizing the importance of mimicking the native properties and functions of cells to best exploit their use for biomedicine and bioengineering.
